# Pharmacological Exploration of Traditional Chinese Medicine and Tujia Ethnomedicine in Rheumatoid Arthritis Therapy: From Historical Clinical Wisdom to Contemporary Scientific Inquiry

**DOI:** 10.3390/ph19060937

**Published:** 2026-06-14

**Authors:** Qingling Xie, Jisheng Liu, Wei Su, Jiangyi Luo, Mengying Lyu, Yan Zhao, Yunmei Lan, Ling Liang, Caiyun Peng, Wei Wang, Hanwen Yuan

**Affiliations:** TCM and Ethnomedicine Innovation & Development International Laboratory, School of Pharmacy, Hunan University of Chinese Medicine, Changsha 410208, China; xieql12@126.com (Q.X.); ljs38635@gmail.com (J.L.); suwei0310@163.com (W.S.); jyiluo1998@163.com (J.L.); dancytime@163.com (M.L.); z15675921668@163.com (Y.Z.); 18173087636@163.com (Y.L.); lliang901@stu.hnucm.edu.cn (L.L.); 002142@hnucm.edu.cn (C.P.)

**Keywords:** Traditional Chinese medicine, Tujia ethnomedicine, Rheumatoid arthritis, Signaling pathways, Pharmacology

## Abstract

Rheumatoid arthritis (RA) remains a recalcitrant clinical challenge, as modern therapies are often hampered by adverse effects, suboptimal responses, and failure to achieve radical cure. Traditional Chinese Medicine (TCM) and Tujia ethnomedicine, with centuries of accumulated experience in managing RA (classified as “Bi Syndrome” in TCM), offer distinct theoretical frameworks and abundant therapeutic resources. TCM emphasizes syndrome differentiation-based holistic regulation, while Tujia ethnomedicine relies on indigenous medicinal plants and empirically derived therapies shaped by its unique geographical context. This review aims to accelerate the integration of traditional wisdom with contemporary pharmacology for the development of novel RA therapies. A comprehensive literature search was performed across PubMed, Web of Science, CNKI, and ethnomedical monographs to synthesize data on their theoretical underpinnings, therapeutic strategies, mechanisms of action, and clinical efficacy. TCM and Tujia ethnomedicine possess significant anti-RA effects, characterized by multi-component, multi-target synergistic mechanisms that complement modern medicine. However, they face common challenges including unclear material bases of active components, insufficient standardized clinical evidence, and inadequate quality control protocols. This review provides a critical foundation for integrating TCM/Tujia ethnomedicine with modern pharmacology, highlighting the urgent need for further research to clarify active constituents, establish standardized protocols, and validate clinical efficacy—ultimately facilitating the development of safer, more effective RA therapies.

## 1. Introduction

Rheumatoid arthritis (RA) is a systemic autoimmune disorder characterized by symmetric polyarthritis that progresses to irreversible cartilage and bone destruction, culminating in joint deformity and functional impairment [1]. Global meta-analyses report a prevalence of 0.5–1.0%, with a female-to-male ratio of 3:1 [2,3]. The absolute disease burden is projected to rise continuously, driven by population ageing, evolving environmental exposures, and enhanced detection of early-stage disease. Modern medicine has advanced RA treatment, with options including non-steroidal anti-inflammatory drugs (NSAIDs), glucocorticoids (GC), and disease-modifying anti-rheumatic drugs (DMARDs) [4]. However, long-term administration of these agents is associated with side effects, including gastrointestinal injury and hepatorenal impairment [5]. Additionally, certain drugs exhibit delayed onset of action or reduced efficacy in the later course, and they fail to achieve a radical cure, necessitating long-term maintenance therapy [5,6].

Traditional Chinese medicine (TCM), with an over 2000-year history, unique theories, long clinical experience, and diverse natural resources, demonstrates remarkable advantages in regulating overall bodily functions and offering complementary RA management strategies [7]. TCM uses syndrome differentiation-based holistic treatment to restore bodily balance, reducing inflammation, protecting joints, and minimizing toxicity [8]. For example, the root of *Tripterygium wilfordii* Hook. f. is traditionally used to dispel wind–dampness, unblock collaterals, subdue swelling, and relieve pain. Through multiple constituents such as wilforlide, celastrol, triptolide, and glycosides, the herb inhibits the release of inflammatory mediators, rebalances immune cells, and attenuates destruction of articular cartilage via modulation of the nuclear factor-κB (NF-κB), phosphatidylinositol 3-kinase/protein kinase B (PI3K/AKT), and Janus kinase/signal transducer and activator of transcription (JAK/STAT) signaling pathways, thereby producing its anti-rheumatoid arthritis (RA) action [9,10]. Tujia ethnomedicine, a branch of Chinese ethnomedicine from the cold-humid Wuling Mountain Area (a high-risk region for RA), has been refined by Tujia people over generations [11]. Guided by the “Three Elements Theory” and “Toxin Theory,” it uses local herbs and unique therapies for RA [12]. In-depth research into the application of both in RA treatment helps improve clinical efficacy and promotes the inheritance and innovation of traditional medicine, holding significant implications for advancing global medical development.

This review systematically summarizes TCM/Tujia ethnomedicine’s theoretical understanding of RA (compared with modern medicine’s pathogenesis), elaborates on their therapeutic strategies and mechanisms, discusses current challenges in their application/modernization, and proposes future research directions to integrate traditional and modern medicine for RA. By bridging time-honored clinical wisdom and cutting-edge investigation, the review aims to serve as a definitive reference for future RA research and to foster the rational development, evidence-based validation, and global acceptance of TCM and Tujia ethnomedicine in RA treatment. A literature search was conducted in PubMed, Web of Science, CNKI, Google Scholar, and ethnomedical monographs using terms related to RA, TCM, Tujia ethnomedicine, Bi syndrome, herbal medicine, pharmacology, clinical application, and safety. Studies were selected if they addressed traditional theory, ethnomedicinal application, active constituents, pharmacological mechanisms, clinical evidence, or safety of TCM/Tujia ethnomedicine in RA. Studies unrelated to RA, duplicate publications, and reports lacking clear pharmacological, clinical, or ethnomedical relevance were excluded. The quality and limitations of the included studies were qualitatively considered based on the information reported in each study, including study design, sample size, control setting, randomization and blinding, follow up duration, outcome measures, adverse event reporting, reproducibility, and potential sources of bias where applicable. Evidence from in vitro and animal studies was interpreted as preclinical evidence and was not directly extrapolated to clinical efficacy.

## 2. Understanding of RA in Traditional and Modern Medical Systems

### 2.1. The Interpretation of RA Based on Bi Syndrome in TCM

**Figure 1:**
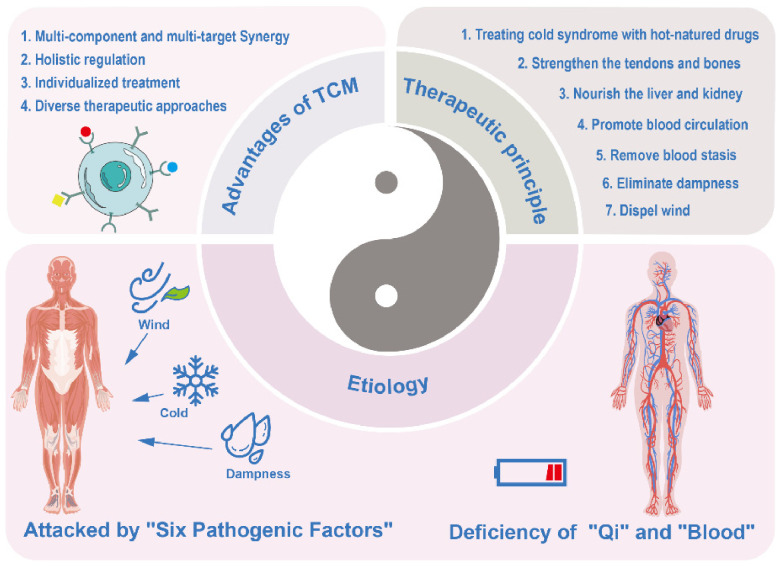
TCM Theory related to RA.

According to TCM theory, six natural phenomena termed the “Six Pathogenic Factors” (wind, cold, summer-heat, dampness, dryness, fire) can cause exogenous diseases when invading the body [13]. RA in modern medicine corresponds to Bi Syndrome (arthromyodynia) in TCM, a classic exogenous disorder caused by the combined attack of wind, cold, and dampness evils on the joints, muscles, and bones (Figure 1) [14]. Cold and damp environments facilitate the onset and progression of RA, which is consistent with patients’ common clinical manifestations of aversion to cold and preference for warmth [15,16].

RA initially presents as a cold syndrome due to cold evil, but may transform into a heat syndrome over time. This dynamic evolution necessitates strict adherence to the fundamental TCM principle of “treating cold syndromes with hot-natured herbs and heat syndromes with cold-natured herbs” [16]. Ancient physicians emphasized qi–blood deficiency as a root cause of RA, with the liver (storing “Blood”) and kidney (governing “Qi”) being critical [17]. Thus, therapies often combine herbs to tonify the liver–kidney, strengthen muscles and bones, activate blood, and dispel wind-dampness [17].

TCM practitioners exhibit diverse insights and therapeutic strategies for RA, exemplifying the core characteristics of syndrome differentiation-based treatment and individualization. The multi-component, multi-target synergistic action of TCMs represents a distinct advantage over single-target chemical drugs, alongside their relatively low toxicity profile [18]. Beyond oral administration, TCM external therapies, such as acupuncture, tuina (Chinese holistic massage), moxibustion, and cupping, offer unique benefits for RA [15,19]. However, these interventions remain underutilized in clinical practice due to insufficient depth and breadth of research, as well as the lack of standardized treatment protocols.

### 2.2. Tujia Ethnomedical Understanding and Therapeutic Principles for RA

In the Wuling Mountain Area inhabited by the Tujia ethnic group, the towering, rugged terrain and cold–humid climate serve as significant predisposing factors for RA. In this geographically isolated environment, Tujia ethnomedicine has evolved a unique theoretical framework centered on the “Three Elements Theory” [11]. Inspired by the TCM theory of “six pathogenic factors causing diseases”, Tujia Ethnomedicine has developed the “Toxin Theory”, which posits that cold-damp toxins interact synergistically with wind-toxins, migrating to joints to obstruct “Qi-Blood” circulation and induce RA [12].

Historically lacking a native written language, Tujia ethnomedical knowledge has relied primarily on oral transmission of clinical experience, resulting in limited theoretical and systematic elaboration [20]. Although rich practical experience in RA treatment exists, a standardized diagnostic and therapeutic system of Tujia ethnomedicine remains undeveloped, and the mechanistic basis of many characteristic therapies requires further exploration [21].

### 2.3. The Pathogenesis of RA in Modern Medicine System

**Figure 2:**
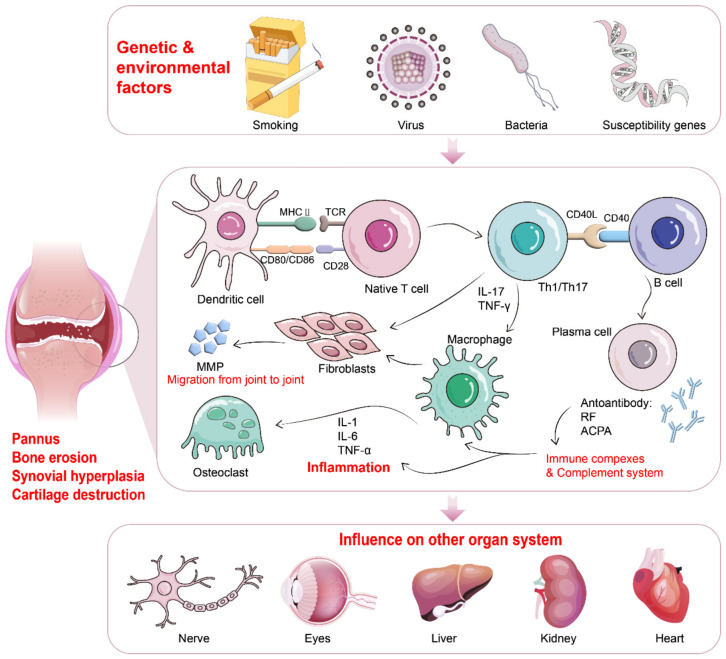
The pathogenesis of RA.

RA is a complex autoimmune disease caused by the interaction of multiple factors, including genetics, environment, immune abnormalities, and epigenetics, with genetic factors contributing approximately 60% to its etiology [22]. Risk genes such as HLA-DRB1 shared epitope alleles (*01 and *04) and PTPN22 increase susceptibility by regulating antigen presentation and immune cell signaling [23]. Environmental factors (e.g., smoking, bacterial and viral infections, diet, and hygiene practices) can induce protein citrullination and activate innate immunity, synergizing with genetic factors to disrupt immune tolerance (Figure 2) [23,24].

Dendritic cells (DCs) may migrate to inflamed synovial tissues and secrete IL-12 and IL-23, driving the activation of Th1/Th17 cells [25]. These activated cells release pro-inflammatory mediators (TNF-α, IL-17, IFN-γ, and RANK-L), which in turn activate macrophages and fibroblasts [26]. Activated macrophages secrete potent pro-inflammatory cytokines (TNF-α, IL-1β, and IL-6), maintaining the inflammatory microenvironment in the synovium [27]. Synovial fibroblasts (FLS) acquire an invasive phenotype and secrete matrix metalloproteinases (MMPs) and RANK-L, collaborating with immune cells to degrade cartilage and bone tissues [26]. The binding of CD40 ligand (CD40L) on Th cells to CD40 on B cells provides co-stimulatory signals, which, together with IFN-γ and TNF-α secreted by Th1/Th17 cells, promote B cell differentiation into plasma cells/plasmablasts. This process generates anti-citrullinated protein antibodies (ACPA) and rheumatoid factor (RF), forming immune complexes that activate the complement system and further amplify autoimmune responses [1]. The regulatory network composed of these cytokines and chemokines collectively drives synovial inflammation, pannus formation, bone destruction, and joint deformity, establishing a vicious cycle.

In addition, the abnormal immune response in RA patients mediates multi-organ damage through cytokines and autoantibodies, such as increasing the risk of myocardial infarction, triggering iritis, renal amyloidosis, interstitial lung disease, and peripheral neuropathy [23,28]. Therefore, early targeted intervention of inflammatory pathways such as TNF-α and IL-6 is important for controlling disease progression and improving clinical prognosis in RA patients.

**Figure 3:**
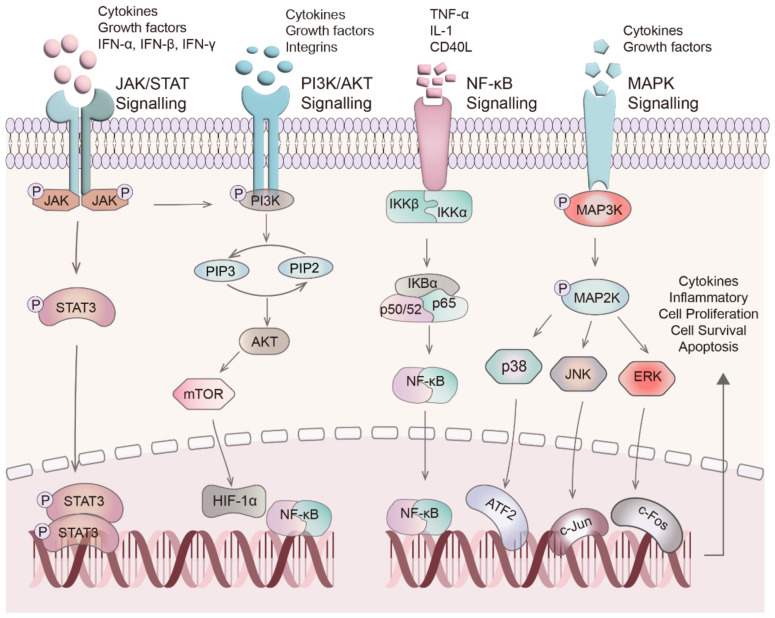
Main signaling pathways related to RA.

The occurrence and development of RA involve complex interactions among multiple signaling pathways, including JAK/STAT, MAPK, NF-κB, and PI3K/AKT/mTOR (Figure 3) [28,29]. Cytokines, such as IL-6 and TNF-α, can activate JAK1/2/3 kinases, which may induce RANKL expression and inhibit OPG by phosphorylating transcription factors like STAT3 [30]. This process may contribute to osteoclast differentiation and bone resorption while regulating the abnormal proliferation of synovial fibroblasts and the secretion of inflammatory cytokines. PI3K catalyzes PIP2 to generate PIP3 for AKT activation, regulating cell proliferation, autophagy, and osteoclast differentiation through the mTOR pathway [31]. Abnormal activation of this pathway may promote synovial cell hyperplasia, inflammatory cytokine release, and participates in bone erosion and angiogenesis. Activated by TNF-α and IL-1β, NF-κB can induce the expression of pro-inflammatory cytokines, forming a positive feedback loop of inflammation that exacerbates synovial inflammation, angiogenesis, and osteoclast differentiation, leading to aggravated articular bone erosion and tissue damage [32]. The MAPK pathway comprises three main subfamilies (ERK, JNK, and p38), which inflammatory cytokines can activate to promote synovial inflammation and cartilage destruction via regulating the production of pro-inflammatory cytokines (e.g., IL-6, TNF-α) and MMP expression [33,34]. These pathways may interact with each other and contribute to inflammatory imbalance and bone metabolic disorder in RA. Targeted interventions (e.g., JAK inhibitors, mTOR inhibitors), by blocking key signaling nodes, demonstrate potential to inhibit joint destruction, providing an important theoretical basis for precision therapy of RA.

The involvement of multiple interacting pathways provides a basis for comparing modern targeted therapies with the potential regulatory characteristics of TCM and Tujia ethnomedicine. Modern biologics, such as TNF-α inhibitors, mainly act by blocking a defined cytokine pathway and have established clinical value in RA treatment [4]. However, RA pathogenesis involves multiple immune cells, cytokines, and signaling pathways, including NF-κB, JAK/STAT, MAPK, and PI3K/AKT/mTOR [28,29]. In this context, TCM formulas and Tujia ethnomedicinal preparations may provide complementary value by regulating several inflammatory and immune processes, including cytokine production, macrophage activation, synovial fibroblast proliferation, osteoclast differentiation, and cartilage matrix degradation [8]. This potential multi-target regulation may partly explain the pharmacological synergy of traditional medicines and may provide complementary value in complex or heterogeneous inflammatory conditions. However, it should not be interpreted as clinical superiority over biologics, and its relevance to inadequate response or secondary loss of response to targeted therapies requires further validation.

## 3. TCM Phytomedicines and Herbal Therapies for RA

### 3.1. Classic Formulas for Treating RA

In clinical practice, TCM formulas are mostly used as compound prescriptions, formulated by combining two or more herbs in specific proportions. Guided by the TCM “holistic concept” and following the “Jun-Chen-Zuo-Shi” (monarch-minister-assistant-guide) principle, these prescriptions target the core pathogenesis of diseases while maintaining the overall balance of qi, blood, yin, and yang in the human body, thus comprehensively regulating physiological functions. Compared with single herbs, compound prescriptions have more complex chemical compositions, making research on their mechanisms of action more challenging and resulting in a relatively weak foundation for relevant studies. Although increasing studies have investigated the pharmacological activities and signaling pathways of classical formulas in RA, the strength of evidence remains uneven. Some formulas have been evaluated in clinical studies, whereas many mechanistic interpretations are still mainly derived from in vitro experiments or animal models. For example, Duhuo Jisheng Decoction is supported by a meta-analysis (42 RCTs), and Yishen Tongbi Decoction has been evaluated in a double-blind non-inferiority RCT. In contrast, evidence for several other formulas is mainly derived from animal models or cell based experiments. Therefore, their potential therapeutic relevance should be discussed in relation to the corresponding evidence type, study design, sample size, follow up duration, adverse event reporting, and reproducibility, rather than being directly extrapolated to confirmed clinical efficacy.

Duhuo Jisheng Decoction is a classic anti-RA compound prescription. Composed of 15 TCMs, it is traditionally used for dispelling wind–dampness, relieving arthralgia, tonifying the liver and kidney, and replenishing qi and blood. Modern pharmacological studies have suggested that it may inhibit the expression of pro-inflammatory factors by regulating signaling pathways such as PI3K/AKT and NF-κB, thereby contributing to potential anti-inflammatory and analgesic effects (Table 1) [35,36]. Preclinical studies have reported that Wutou Decoction may alleviate inflammatory responses in experimental arthritis. It inhibited the HMGB1/NF-κB pathway through SIRT1-mediated deacetylation, reduced M1 macrophage polarization and the release of inflammatory factors, and regulated the JAK2/STAT3 pathway to maintain Treg/Th17 balance [37,38,39]. Additionally, the herbs in the prescription may exert a synergistic effect to enhance their therapeutic efficacy [37]. Studies in animal models and RA-FLS suggest that Ermiao San/Wan may reduce inflammatory responses and RA-FLS migration and invasion, possibly involving the PI3K/AKT/mTOR/HIF-1α signaling pathway. Specifically, it downregulated the expression of glycolysis-related proteins (Glut1 and HK2), reduced the migration and invasion of RA-FLS, and decreased the release of IL-6 and TNF-α [40]. Ermiao Wan (a pill formulation of Ermiao San) reduced the activity of COX-2, mPGES-1, and ALOX5 in the arachidonic acid metabolism pathway and the activity of XDH in the purine metabolism pathway. This further led to decreased expression of inflammatory factors (e.g., IL-1β, IL-6), tissue injury factors (e.g., MMP-3, CRP), and rheumatoid factors (e.g., CCP-Ab, RF), thereby suggesting potential anti-inflammatory effects and restoring immune and skeletal functions [41].

Yishen Tongbi Decoction suppressed the SLC3A2/integrin β3 signaling pathway and its downstream proteins (p-FAK, p-Src) to inhibit the proliferation and migration of RA-FLS, and activated the FcγRIIb/Lyn/SHP-1 pathway while inhibiting CD19 phosphorylation and intracellular Ca^2+^ influx to reduce excessive B cell activation [42]. In CIA mice, Yishen Tongbi Decoction dose-dependently attenuated paw swelling and synovial inflammation, preserved cortical and trabecular bone micro-architecture, and achieved a significantly earlier onset of action than MTX [43]. A double-blind non-inferiority RCT reported that Yishen Tongbi Decoction exhibited non-inferiority to MTX in the treatment of active RA, achieved a superior short-term response, and maintained a favorable safety profile with acceptable adverse events [44]. Nevertheless, this clinical evidence should be interpreted with attention to trial design, sample size, follow up duration, reporting of randomization and blinding, adverse event monitoring, and reproducibility. Further multicenter studies are needed to verify its long-term efficacy and safety.

In clinical practice, other classic TCM formulas, such as Juanbi Decoction [45], Baihu Jia Guizhi Decoction [46], Guizhi Shaoyao Zhimu Decoction [47], and other formulas, were also employed for the treatment of RA (Table 1). The core of TCM treatment lies in syndrome differentiation and treatment. Clinicians would make individualized adjustments to the basic formulas based on the specific syndrome types of RA patients. Taking Duhuo Jisheng Decoction as an example, doctors would flexibly add or remove herbs or adjust dosages based on the original formula’s composition principle according to the different clinical manifestations of patients, so that the formula was better adapted to the patient’s current condition, thereby potentially improving the therapeutic effect [48]. These examples indicate the flexibility of classical formulas in individualized RA management. However, future clinical studies should adopt standardized intervention protocols, appropriate control settings, adequate sample sizes, transparent reporting of randomization and blinding, complete adverse event reporting, and reproducible outcome assessment.

### 3.2. Chinese Patent Medicines for RA Treatment

Classic formulas were processed into Chinese patent medicines (e.g., tablets, capsules, granules), with the benefits of convenient administration, accurate dosage, and controllable quality. These formulations may be more adaptable to modern clinical needs, facilitated long-term administration for maintaining RA stability, and this also reflected TCM modernization. According to the Clinical Application Guidelines for Chinese Patent Medicines in the Treatment of Rheumatoid Arthritis (2022, National Administration of Traditional Chinese Medicine), 17 main Chinese patent medicines were identified for RA treatment [49].

Kunxian Capsules reduced the levels of IL-8 and interferon-γ-inducible protein 10 (γIP-10) in the peripheral blood of CIA rats [50]. Clinical studies reported that t Kunxian Capsules may alleviate symptoms in RA patients, improved their joint function and mobility, and show potential synergistic effect when co-administered with MTX [51,52]. However, because some clinical evidence is based on combination therapy or clinical observation, the independent contribution of Kunxian Capsules and the risk of bias should be interpreted with caution. More rigorously designed trials with clear randomization, blinding, allocation concealment, standardized outcome measures, and adverse event reporting are needed. Tripterygium Glycoside Tablets, the primary clinical preparation of *Tripterygium wilfordii*, have shown potential therapeutic benefits in RA in clinical and experimental studies [53,54,55]. The anti-RA effects of *Tripterygium wilfordii* total glycosides and its major components (e.g., diterpenoids, triterpenoids, sesquiterpene alkaloids) have been investigated in multiple studies [9,56]. Among these constituents, triptolide and celastrol have been relatively well characterized in relation to RA mechanisms. Triptolide has been linked to inflammatory cytokines, NF-κB, COX-2, MMPs, and RANK/RANKL/OPG signaling, whereas celastrol has been associated with NF-κB, MAPK, JAK/STAT, and TLR4/MD2 signaling in preclinical RA models [9,10]. Despite clinical application since 1984, Tripterygium Glycoside Tablets still require further basic research, particularly on efficacy-toxicity relationships, active constituents, and safety control [57]. Simiao Pill, derived from the classic Ermiao San, was formulated by adding Yiyiren (seeds of *Coix lacryma-jobi*) and Niuxi (roots of *Achyranthes bidentata*) to the base prescription, which enhanced its effects of draining dampness and relieving bi syndrome. It showed advantages in treating damp–heat-induced lower limb Bi pain and joint dysfunction, and was more suitable for severe RA. Simiao Pill significantly reduced joint swelling and arthritis index in CIA mice and downregulated mRNA expression of TNF-α, IL-6, and IL-1β in AA rats. Its therapeutic mechanism was closely associated with the JAK2/STAT3 and TGF-β/SMAD2/3 signaling pathways [58,59]. Additionally, clinical studies revealed that when combined with MTX, Simiao Pill notably improved RA-related symptoms such as joint swelling, pain, morning stiffness, and deformity [60].

In addition, some Chinese patent medicines for RA treatment have been developed into multiple dosage forms. For example, Zhengqing Fengtongning was available in conventional tablets and sustained-release tablets. Sinomenine, a major alkaloid from *Sinomenium acutum* (Qingfengteng), is the main active constituent of Zhengqing Fengtongning and has been reported to be involved in the regulation of PI3K/Akt signaling, inflammatory responses, RA FLS migration and invasion, and immune cell differentiation in RA models [61]. Although Zhengqing Fengtongning differs from many compound prescriptions in having sinomenine as its main active constituent, it is still classified as a Chinese patent medicine in the *Pharmacopoeia of the People’s Republic of China*. Together with triptolide and celastrol, sinomenine provides an example of how selected dominant constituents can be linked to reported molecular targets or pathway nodes. However, for most compound prescriptions, such relationships remain only partially characterized because each herb contains multiple constituents and the overall pharmacological effect may involve several inflammatory and immune pathways. Further studies are needed to clarify dominant constituents, molecular targets, dose response relationships, and synergistic mechanisms. This review further summarizes other major Chinese patent medicines used for RA treatment (Table 2). The available evidence supporting these medicines differs in study design and clinical relevance. Tripterygium Glycosides Tablet is supported by a meta-analysis (40 RCTs), whereas Biqi Capsule and Wangbi Tablet/Capsule have been evaluated in randomized clinical studies. For several other preparations, the available evidence is mainly derived from animal models, cell based experiments, clinical observations, or studies involving concomitant conventional RA therapies, which limits direct attribution of efficacy to the Chinese patent medicine alone. Therefore, conclusions about clinical efficacy should be interpreted in relation to study design, sample size, control setting, concomitant medication, adverse event reporting, potential bias, and reproducibility. Findings from animal and cell based studies should be considered preclinical evidence rather than direct proof of clinical efficacy.

Current research on anti-RA Chinese patent medicines generally faces the following problems: (1) Although these medicines had a long history of clinical application, their basic research remains relatively insufficient, and their specific mechanisms of action needed further analysis. (2) Most studies remained at the level of clinical observation, and the medicines were often used in combination with classic chemical drugs. The lack of multi-center, large-sample, and well-controlled studies in-depth studies led to insufficient persuasiveness of efficacy evidence. (3) Existing studies focused on single TCMs or their main components, while the formula rules of compound preparations and the specific role of each medicinal material in the products remained unclear. (4) The raw medicinal materials used in these medicines were of various types; some had multiple origin sources, and a few were not included in the Pharmacopoeia of the People’s Republic of China, so their quality control systems needed further improvement.

### 3.3. Safety Considerations for TCM Therapies in RA Treatment

Although TCM formulas and Chinese patent medicines may provide complementary options for RA management, their safety profiles require careful evaluation, particularly for preparations containing herbs with a narrow therapeutic window. This concern is especially relevant to *Tripterygium* related preparations, such as Tripterygium Glycoside Tablets and Kunxian Capsules, and formulas containing *Aconitum* species, such as Wutou Decoction and Panlongqi Tablet. Among these preparations, *Tripterygium wilfordii* and related products have been widely investigated for RA and other autoimmune diseases, but their clinical use is limited by potential toxicity and individual differences in tolerance [61]. Reported adverse effects include gastrointestinal reactions, hepatic and renal injury, reproductive toxicity, hematological abnormalities, cutaneous reactions, and immunosuppression [62]. Therefore, safer use requires standardized dosage, appropriate treatment duration, careful patient selection, and regular monitoring of liver and kidney function, blood routine indices, and reproductive risk.

Variability in herbal materials and preparations is another key factor affecting both efficacy and safety. Many RA related formulas and Chinese patent medicines contain multiple herbs and are prepared as decoctions, tablets, capsules, granules, or sustained release formulations. Differences in botanical origin, harvesting period, processing method, extraction procedure, and dosage form may alter chemical composition, pharmacological activity, and toxicity risk. For formulas containing Aconitum species, traditional processing is essential for toxicity reduction, but processing parameters, residual toxic alkaloids, and clinical dosage must be strictly controlled [63]. Chemical marker based quality control, batch consistency evaluation, and pharmacokinetic studies are therefore needed to improve reproducibility and safety.

Interactions between TCM preparations and standard RA therapies also require attention because many patients use Chinese patent medicines together with methotrexate, glucocorticoids, NSAIDs, or biological agents. Such combinations may offer complementary benefits, but they may also increase the risk of hepatotoxicity, nephrotoxicity, gastrointestinal adverse events, or excessive immunosuppression [64]. Future studies should systematically evaluate both pharmacokinetic and pharmacodynamic interactions in combination therapy. For highly active but toxic constituents such as triptolide, improved formulations and local delivery strategies, including transdermal microneedle delivery, may help reduce systemic exposure, although these approaches still require further pharmacological, toxicological, and clinical validation [65]. Overall, rigorous safety assessment, standardized adverse event reporting, and post approval surveillance are essential before broader clinical translation.

**Table 2:** Chinese patent medicines for RA treatment and their research status.

No.	Drugs	Composition	Traditional Use	Evidence Type	Pharmacological Effect	Signal Pathway	Reference
1	Kunxian Capsule (昆仙胶囊)	Kunming Shanhaitang (昆明山海棠), Yingyanghuo (淫羊藿), Gouqizi (枸杞子), Tusizi (菟丝子)	Tonifying the kidney and dredging collaterals; Dispelling wind and eliminating dampness	CIA rats; Clinical observation	IL-8, γIP-10 ↓	-	[50,51,52]
2	Tripterygium Glycosides Tablet (雷公藤多苷片)	Tripterygium Glycosides (雷公藤多苷)	Dispel wind and detoxify; Eliminate dampness and reduce swelling; Relax muscles and unblock collaterals	CIA rats; meta-analysis (40 RCTs)	TNF-α, IL-6, VEGF, TGF-β1, IL-1β ↓	NF-κB; MAPK	[54,55]
3	Simiao Tablet (四妙片)	Huangbai (黄柏), Cangzhu (苍术), Niuxi (牛膝), Yiyiren (薏苡仁)	Clear heat and drain dampness; Free the sinews and relieve bi syndrome	CIA mice; AA rats; Clinical observation	TNF-α, IL-6, IL-1β↓	JAK2/STAT3; TGF-β/SMAD2/3	[58,59,60]
4	Zhengqing Fengtongning Tablet (Sustained Release Tablet) (正清风痛宁片/缓释片)	Sinomenine (青藤碱)	Dispel wind and eliminate dampness; Activate blood and dredge collaterals; Promote diuresis and reduce swelling	CIA rats meta-analysis appraisal	RANKL, MMPs ↓	-	[66,67]
5	Biqi Capsule (痹祺胶囊)	Maqianzi (马钱子), Dilong (地龙), Dangsheng (党参), Fuling (茯苓), Gancao (甘草), Chuanxiong (川芎), Danshen (丹参), Sanqi (三七), Niuxi (牛膝)	Invigorate qi and nourish blood; Dispel wind and eliminate dampness; Activate blood circulation and relieve pain	MH7A cells; CIA rats; Clinical observation	IL-18, TNF-α, IL-6, IL-1β ↓	JAK/STAT	[68,69,70,71]
6	Yishen Juanbi Pill (益肾蠲痹丸)	Gusuibu (骨碎补), Dihuang (地黄), Danggui (当归), Xuchangqing (徐长卿), Tubiechong (土鳖虫), Wugong (蜈蚣), Quanxie (全蝎), Fengfang (蜂房), Dilong (地龙), Wushaoshe (乌梢蛇), Yanhusuo (延胡索), Luxiancao (鹿衔草), Yingyanghuo (淫羊藿), Xungufeng (寻骨风), Laohecao (老鹳草), Jixueteng (鸡血藤), Lvcao (律草), Huzhang (虎杖)	Warm and tonify kidney Yang; Benefit kidney and strengthen governor vessel; Search for and expel wind pathogens; Relieve Bi syndrome and dredge collaterals.	Osteoclast and Treg cells; CIA rats; Clinical observation	IL-10 ↓	JAK/STAT	[72,73,74]
7	Wangbi Tablet (Capsule) (尪痹片/胶囊)	Dihuang (地黄), Xuduan (续断), Fuzi (附子), Yingyanghuo (淫羊藿), Weilingxian (威灵仙), Zaoci (皂刺), Yangu (羊骨), Zhimu (知母), Shenjincao (伸筋草), Honghua (红花), Duhuo (独活), Baishao (白芍), Gusuibu (骨碎补), Guizhi (桂枝), Fangfeng (防风), Goujizi (枸杞子)	Nourish the liver and kidney; Strengthen the muscles and bones; Dispel wind and dampness; Dredge the meridians and collaterals	CIA rats; multicenter double-blind RCT	TNF-α, IL-6, IL-17A, IL-1α, IL-1β, IL-2, IL-12P70, IFN-γ, G-CSF ↓	cGAS-STING; Wnt/β-catenin	[75,76,77]
8	Yuxuebi Tablet (Capsule, Granule) (瘀血痹片/胶囊/颗粒)	Ruxiang (乳香), Weilingxian (威灵仙), Honghua (红花), Danshen (丹参), Moyao (没药), Chuanniuxi (川牛膝), Chuanxiong (川芎), Danggui (当归), Jianghuang (姜黄), Xiangfu (香附), Huangqi (黄芪)	Promote blood circulation to remove blood stasis; Dredge collaterals to relieve pain	RA-FLS cells CIA rats; Clinical observation	IL-1β, IL-8, Ras, Raf-1 ↓	Ras/Raf-1/NF-κB; SUCNR1/HIF-1α/TRPV1axis	[78,79,80]
9	Panlongqi Tablet (盘龙七片)	Wujiapi (五加皮), Panlongqi (盘龙七), Duzhong (杜仲), Zhuangjindan (壮筋丹), Zhuzishen (珠子参), Qingwaqi (青蛙七), Guoshanlong (过山龙), Qinjiao (秦艽), Muxiang (木香), Zusima (祖司麻), Luoshiteng (络石藤), Chuanwu (川乌), Baimaoqi (白毛七), Tiebangchui (铁棒锤), Caowu (草乌), Laoshuqi (老鼠七), Zhizhuliao (支柱蓼), Honghua (红花), Moyao (没药), Zhugenqi (竹根七), Xiecao (缬草), Shenjincao (伸筋草), Niuxi (牛膝), Danshen (丹参), Yangjiaoqi (羊角七), Balima (八里麻), Chonglou (重楼), Ruxiang (乳香), Danggui (当归)	Promote blood circulation to remove blood stasis; Dispel wind and eliminate dampness; Reduce swelling and relieve pain	CIA rats; AA mice; HFLS cells; Clinical observation	TNF-α, IL-6, MDA↓ IL-10, SOD ↑	Keap1/Nrf2/HO-1; PI3K/Akt; MAPK; JAK/STAT	[81,82,83]
10	Shirebi Tablet (Capsule, Granule) (湿热痹片/胶囊/颗粒)	Cangzhu (苍术), Rendongteng (忍冬藤), Dilong (地龙), Lianqiao (连翘), Huangbo (黄柏), Yiyiren (薏苡仁), Fangfeng (防风), Chuanniuxi (川牛膝), Fenbixie (粉萆薢), Sangzhi (桑枝), Fangji (防己), Weilingxian (威灵仙)	Dispel wind and eliminate dampness; Clear heat and reduce swelling; Dredge collaterals and relieve pain	AIA rats	TNF-α, IL-6, VEGF ↓ IL-10, SOD ↑	-	[84]
11	Xinhuang Tablet (新癀片)	Zhongjiefeng (肿节风), Sanqi (三七), Niuhuang (牛黄), Zhudanfen (猪胆粉), Xiaofantianhua (肖梵天花), Zhenzhu (珍珠), Shuiniujiao Nongsuofen (水牛角浓缩粉), Hongqu (红曲), Indometacin (吲哚美辛)	Clear heat and detoxify; Promote blood circulation to remove blood stasis; Reduce swelling and relieve pain	Inflammation Model of mice and rats; Clinical observation	IL-1α, IL-1β, HIS ↓; IL-10 ↑	NF-κB	[85,86]

Note: The botanical names of the herbal drugs are provided in Table S1 in the Supplementary Data. RCT, randomized controlled trial.

## 4. Tujia Ethnomedicinal Plants and Remedies for RA

Tujia ethnomedicine embodies the valuable diagnosis and treatment experience accumulated by the ancestors of the Tujia people in their long-term production and daily life. However, restricted by factors such as historical inheritance models and regional development conditions, the construction of systematization and standardization for the theoretical system of Tujia ethnomedicine remains weak. It especially lacks mature core theoretical support, such as drug property compatibility and the “monarch, minister, assistant, and guide” theory. This deficiency directly limits the scientific compatibility research and compound drug development of Tujia ethnomedicine. As a result, in folk practice, most of its medicines are still used directly in the form of single herbs. The common processing methods are only simple means, such as mashing fresh herbs for external application and decocting them in water for internal use. In the industrialization development and transformation process, Tujia ethnomedicine also faces significant bottlenecks. Few medicines can break through the limitation of single-herb application, complete development in accordance with modern pharmaceutical standards, and finally be transformed into proprietary Chinese patent medicine products that meet clinical needs.

The research group has conducted in-depth studies on the chemical constituents and anti-RA mechanism of two Tujia ethnomedicines, including Xuetong (the stems of *Kadsura heteroclite*) and Heilaohu (the roots of *Kadsura coccinea*). Over 300 components have been isolated from the two herbs, with triterpenoids, lignans, and sesquiterpenoids identified as the major classes [87,88,89]. The ethanol extract of Xuetong and its principal component, xuetongsu have shown potential anti-inflammatory, analgesic, and anti-arthritic effects in preclinical models. These effects may involve NF-κB and JAK/STAT signaling, thereby contributing to the downregulation of inflammatory mediators [90,91,92]. Specifically, these interventions were reported to attenuate synovial hyperplasia, pannus formation, cartilage destruction, and bone erosion in AIA rats. Sesquiterpenoids and triterpenoids isolated from Heilaohu, such as heilaohuacid D and Gaultheriadiolide, could inhibit the expression of TNF-α and IL-6 in LPS-induced RAW 264.7 cells via the NF-κB and JAK2/STAT3 signaling pathways [93,94]. However, their anti-RA efficacy in in vivo and clinical settings requires further investigation. Total saponins of the roots of Panax japonicus (Zhujieshen) exerted anti-RA effects by inhibiting glycolysis in M1 macrophages and modulating the balance of colonic content and mucosal microbiota [95]. In clinical practice, a hospital preparation named Compound Zhujieshen Tablets, with Zhujieshen as the principal ingredient, was also used for the treatment of RA.

A systematic summary was made of the Tujia ethnomedicines reported for anti-RA activity (Table 3). Current evidence indicates that the study of Tujia ethnomedicine is gradually shifting from traditional empirical use toward phytochemical characterization and pharmacological validation. However, fundamental research remains limited, and most available studies are still based on preliminary cell or animal experiments. Although over 300 remedies used in folk for RA treatment had been documented through field surveys, standardized basic research, quality control, and clinical validation had not been conducted for most of them [96]. Consequently, their pharmacological activity, mechanism of action, safety, and efficacy remained undefined, and the modernization of Tujia ethnomedicine was severely impeded. Therefore, bioactive constituents needed to be identified, mechanisms elucidated, and rigorous clinical evaluations performed to provide a scientific basis for the development and therapeutic application of anti-RA Tujia ethnomedicines. Future studies should prioritize the standardization of herbal species, medicinal parts, preparation procedures, dosage, quality markers, pharmacological models, safety assessment, and clinical outcome measures. This would help transform Tujia ethnomedicinal practices from oral and empirical traditions into reproducible therapeutic resources for RA supported by scientific evidence.

## 5. Special Therapeutic Approaches for RA in TCM and Tujia Ethnomedicine

In addition to conventional pharmacological treatment, TCM and Tujia ethnomedicine have developed distinctive therapeutic approaches for the management of RA, particularly for relieving pain, stiffness, swelling, and functional limitation. Acupuncture interventions, including manual acupuncture, electroacupuncture, warm needle therapy, and fire needle therapy, have been investigated as adjunctive approaches for RA and may improve pain, disease activity, and inflammatory indicators [110,111]. Moxibustion has also been reported to relieve RA related pain and reduce disease activity, possibly through the regulation of inflammatory mediators [112]. Compared with systemic pharmacological treatment, these external or non-pharmacological interventions are mainly used as complementary approaches rather than independent therapies. In addition, the combination of TCM fumigation and Tuina has been applied in patients with early-stage RA and was reported to improve related symptoms and reduce inflammatory factors [113]. However, the available clinical evidence for these approaches remains limited, and differences in intervention methods, acupoint selection, treatment duration, outcome measures, and study quality should be considered when interpreting their effects.

In the Tujia ethnomedical system, therapies such as external application of fresh herbal juice, fumigation therapy, and Ganyou Huoliao (赶油火疗; i.e., repeatedly dipping the hand in heated tung oil, followed by smearing and pressing the affected area until the skin turns red) have been commonly used for RA related symptoms [114]. These methods are generally intended to dispel cold and dampness, promote local circulation, relieve pain, and improve joint mobility within the traditional Tujia medical framework. However, their mechanisms, efficacy, and safety remain insufficiently validated. To support future clinical evaluation, external Tujia therapies should be standardized in terms of herbal source, medicinal part, preparation method, dosage, application site, treatment frequency, and treatment duration. For Ganyou Huoliao, additional parameters, including tung oil quality, heating temperature, operation procedure, exposure time, skin safety monitoring, contraindications, and clinical outcome measures, should also be clearly defined. Future trials should further adopt clear RA diagnostic criteria, appropriate control groups, standardized outcome measures, adverse event reporting, and reproducible operating protocols. Such standardization would help improve reproducibility and support the transition of these empirical therapies toward clinical evaluation supported by scientific evidence.

## 6. Conclusions and Future Perspectives

Rheumatoid arthritis (RA) is a complex chronic autoimmune disease that continues to pose a major global clinical challenge. Despite substantial advances in modern therapeutics, important unmet needs remain, including treatment-related adverse effects, heterogeneous responses, and limited long-term disease control in some patients. In this context, Traditional Chinese Medicine (TCM) and Tujia ethnomedicine, both rooted in long-term clinical practice, provide distinctive theoretical frameworks and therapeutic resources that may complement contemporary RA management.

TCM interprets RA within the framework of Bi syndrome, emphasizing the involvement of wind, cold, and dampness pathogens, together with underlying deficiencies of the liver–kidney system and qi-blood. Based on syndrome differentiation and individualized treatment, TCM employs multi-component and multi-target interventions, including classical formulas and Chinese patent medicines. Accumulating evidence suggests that these interventions may modulate key signaling pathways, such as NF-κB, JAK/STAT, and PI3K/Akt/mTOR, thereby regulating inflammatory mediator release, attenuating synovial hyperplasia, and alleviating cartilage and bone damage. In addition, external therapies (e.g., acupuncture and fumigation) further expand the therapeutic repertoire of TCM through anti-inflammatory and analgesic effects.

Tujia ethnomedicine, shaped by the ecological and cultural context of the Wuling Mountain area, is characterized by the “Three Elements” and “Toxin” theories and by the extensive use of indigenous medicinal plants and empirically developed remedies for RA-related disorders. Representative herbs such as *Kadsura heteroclita*, *Kadsura coccinea*, and *Panax japonicus*, which contain triterpenoids, lignans, saponins, and other bioactive constituents, have shown anti-RA potential through the regulation of inflammatory and immune-related pathways. In addition, distinctive practices, such as fresh herbal juice external application and Ganyou Huoliao, reflect the practical wisdom of Tujia medicine, although their efficacy, safety, and standardized application still require further scientific validation.

Nevertheless, both TCM and Tujia ethnomedicine face substantial barriers to broader clinical translation and modernization. For TCM, key limitations include insufficient clarification of active constituent/material bases and synergistic mechanisms in compound prescriptions, as well as a lack of high-quality clinical evidence from large, multicenter, standardized studies. For Tujia ethnomedicine, challenges include incomplete theoretical systematization, inadequate standardization of diagnostic and therapeutic protocols, and limited in-depth studies on active components, pharmacological mechanisms, and clinical efficacy. In both systems, variability in raw medicinal materials, insufficient quality control frameworks, and limited industrial transformation further constrain reproducibility and wider application.

Future progress in RA therapy may benefit from a deeper integration of traditional medical knowledge with modern pharmacology and translational research. For TCM, priority should be given to clarifying the active constituent basis and synergistic mechanisms of formulas using modern analytical and systems pharmacology approaches, while strengthening rigorous clinical trials to validate efficacy and safety. For Tujia ethnomedicine, systematic documentation and preservation of oral and practical knowledge should be combined with phytochemical characterization, pharmacological validation, and standardized clinical evaluation to support evidence-based development and modernization.

Furthermore, rational integration of TCM/Tujia ethnomedicine-derived phytomedicines with modern targeted therapies may provide complementary or synergistic benefits, potentially improve therapeutic outcomes while reduce adverse effects. Continued investigation into the crosstalk between traditional therapeutic principles and modern immunological mechanisms may also offer new directions for anti-RA drug discovery and optimized combination strategies. Emerging approaches, including neural networks, machine learning-assisted compound identification, network pharmacology, and omics analyses, may further support the modernization of TCM and Tujia ethnomedicine in RA research. These tools can help prioritize candidate compounds, predict potential targets, interpret multi-component mechanisms, and improve quality control. For Tujia ethnomedicine, AI-assisted LC-MS databases and metabolomic profiling may be useful for identifying characteristic constituents of indigenous medicinal plants. However, these findings should be regarded as exploratory evidence and require pharmacological, toxicological, and clinical validation before clinical application.

In summary, TCM and Tujia ethnomedicine represent valuable sources of natural products, phytomedicines, and therapeutic concepts for RA management. With systematic research, standardized evaluation, improved quality control, and translational validation, these traditional medical systems may contribute meaningfully to the development of safer, more effective, and more individualized RA therapies.

## Figures and Tables

**Table 1:** Research progress of compound prescriptions for RA treatment.

No.	Formula	Composition	Traditional Use	Evidence Type	Pharmacological Effect	Signal Pathway	Reference
1	Duhuo Jisheng Decoction (独活寄生汤)	Duhuo (独活), Xixin (细辛), Rougui (肉桂), Fangfeng (防风), Qinjiao (秦艽), Duzhong (杜仲), Niuxi (牛膝), Sangjisheng (桑寄生), Dihuang (地黄), Shaoyao (芍药), Danggui (当归), Chuanxiong (川芎), Renshen (人参), Fuling (茯苓), Gancao (甘草)	Dispelling wind-damp; Relieving Bi pain; Benefiting liver and kidney; Tonifying Qi and blood.	CIA rats; meta-analysis (42 RCTs)	IL-1β, IL-6, and TNF-α ↓	PI3K/AKT; NF-κB	[35,36]
2	Wutou Decoction (乌头汤)	Chuanwu (川乌), Mahuang (麻黄), Huangqi (黄芪), Shaoyao (芍药), Gancao (甘草)	Warming the meridians and dispelling cold; Eliminating dampness and relieving bi syndrome.	AIA rats; CIA rats; CIA mice; Raw 264.7 cells	IL-1β, IL-6, and TNF-α ↓; IL-10 and TGF-β ↑; Regulating Treg/Th17 balance	NF-κB; JAK2/STAT3	[37,38,39]
3	Ermiao San/Wan (二妙散/丸)	Huangbai (黄柏), Cangzhu (苍术)	Clearing heat and drying dampness	CIA rats; CFA-induced RA rats; RA-FLS	IL-1β, IL-6, IL-17, TNF-α ↓; Tissue injury factors (MMP-3, CRP) and rheumatoid factors (CCP Ab, RF) ↓; Migration and invasion of RA-FLS ↓	PI3K/AKT/mTOR/HIF-1α; Arachidonic acid metabolism; Purine metabolism	[40,41]
4	Yishen Tongbi Decoction (益肾通痹汤)	Kunming Shanhaitang (昆明山海棠), Gouqizi (枸杞子), Nüzhenzi (女贞子), Mohanlian (墨旱莲), Duzhong (杜仲), Danshen (丹参)	Tonifying liver and kidney; Strengthening tendons and bones; Activating blood circulation; Dredging collaterals and relieving pain	HFLS/HFLS-RA cells; CIA mice; CIA rats; double-blind non-inferiority RCT	NO, ROS, TNF-α, IL-1β, IL-6, IL-17, inflammatory cell infiltration, synovial proliferation, joint destruction ↓; TGF-β, IL-10 ↑	SLC3A2/integrinβ3/FAK/Src; JAK/STAT3/SOCS3; FcγRIIb/Lyn/SHP-1	[42,43,44]
5	Juanbi Decoction (蠲痹汤)	Qianghuo (羌活), Duhuo (独活), Guizhi (桂枝), Qinjiao (秦艽), Danggui (当归), Chuanxiong (川芎), Baishao (白芍), Huangqi (黄芪), Fangfeng (防风), Gancao (甘草)	Dispelling wind and eliminating dampness; Relieving bi syndrome and alleviating pain	CIA mice; TNF-Tg mice; MH7A cells	IL-6, IL-8, MMP-1 ↓	NF-κB; p38/JNK-MAPK	[45]
6	Baihu Jia Guizhi Decoction (白虎加桂枝汤)	Shigao (石膏), Zhimu (知母), Rougui (肉桂), Gancao (甘草), Jingmi (粳米)	Clearing heat; Dredging collaterals and relieving pain	AA rats	TNF-α, IL-6 ↓	Ras/MEK/ERK	[46]
7	Guizhi Shaoyao Zhimu Decoction (桂枝芍药知母汤)	Guizhi (桂枝), Shaoyao (芍药), Zhimu (知母), Gancao (甘草), Jingmi (粳米), Mahuang (麻黄), Shengjiang (生姜), Baizhu (白术), Fangfeng (防风), Fuzi (附子)	Activating Yang and relieving Bi syndrome; Dispelling wind and expelling dampness	MH7A cells; CIA rats	IL-6, IL-8, MMP-1, MMP-2, MMP-3 ↓	PI3K/AKT/NF-κB	[47]

Note: The botanical names of the herbal drugs are provided in Table S1 in the supplementary data. In vitro and animal findings were regarded as preclinical evidence. RCT, randomized controlled trial.

**Table 3:** Tujia ethnomedicines used for RA.

No.	Species	Used Parts	Traditional Use	Main Constituents	Experimental	Pharmacological Effect	Signal Pathway	Reference
1	*Kadsura heteroclite* (血筒)	Stems	Dispelling wind and eliminating dampness; Tonifying blood and activating blood circulation; Promoting Qi flow and relieving pain	Triterpenoids, lignans, and sesquiterpenoids.	AIA rats; LPS-induced RAW 264.7; RA-FLS	TNF-α, IL-1β, IL-6, IL-23, IL-17 ↓; IL-10 ↑	JAK2/STAT3; NF-κB	[90,91,92]
2	*Kadsura coccinea* (黑老虎)	Roots and Fruits	Promoting Qi and relieving pain; Dispersing stasis and dredging collaterals	Triterpenoids, lignans, and sesquiterpenoids.	LPS-induced RAW 264.7; RA-FLS cells	TNF-α, IL-6 ↓	JAK2/STAT3; NF-κB	[93,94]
3	*Panax japonicus* (竹节参)	Roots	Activating blood circulation and relieving pain; Reducing swelling and dispelling stasis; Relaxing tendons and dredging collaterals	Saponins	AIA rats LPS-induced RAW 264.7 cells	NO, IL-1β, IL-6 ↓; TGF-β1 ↑	Regulating macrophage polarization and intestinal microbiota	[95]
4	*Schisandra glaucescens* (香血藤)	Stems	Dispelling wind and activating blood circulation; Resolving stasis and reducing swelling	Triterpenoids and lignans,	CIA mice	TNF-α, IL-1β, IL-6 ↓	-	[97,98]
5	*Toddalia asiatica* (百棒七)	Roots and bark	Dispelling wind and eliminating dampness; Activating blood circulation and relieving pain	Alkaloids and coumarins	AIA rats	IL-17A, RORC, IL-1β and IL-6 ↓	Restore the balance of Th17/Treg immune cells	[99]
6	*Aralia echinocaulis* (红槐木/刺老包)	Roots	Nourishing Yin and tonifying kidney; Dispelling wind and eliminating dampness; Strengthening tendons and bones; Dispersing stasis, Reducing swelling and detoxifying	Saponins and flavonoids	AA Rats; RA-FLS cells	Hif-la, p-Akt, Bc1-2 ↓; Caspase-3, Bax ↑	Akt/HIF-1α	[100,101]
7	*Pterocarya hupehensis* (湖北枫杨)	Stem bark	Relieving fever and killing parasites; Dispelling wind and eliminating dampness; Reducing swelling and relieving pain	Flavonoids and phenols	CIA rats	TNF-a, IL-1β, p-p65, Bcl-2 ↓; Caspase-3, Bax ↑	NF-κB	[102,103]
8	*Clematis henryi* (单叶铁线莲)	Root	Clearing heat and detoxifying; Dispelling wind and eliminating dampness; Activating blood circulation and relieving pain	Saponins	CIA rats	NLRP3, Caspase-1, MMP2, MMP9, TNF-α, IL-1β, IL-18 ↓	-	[104]
9	*Ilex centrochinensis* (华中枸骨)	Leaves	Clearing heat and detoxifying; Dispelling wind and eliminating dampness	Flavonoids	MH7A	NO, IL-6, IL-8, IL-1β ↓	-	[105]
10	*Lysionotus pauciflorus* (岩泽兰)	Whole plant	Clearing heat and promoting diuresis; Eliminating phlegm and relieving cough; Activating blood circulation and regulating menstruation	Phenolic acids and flavonoids	AA rats	TNF-α, IL-17, MPP3, IL-6 ↓	-	[106,107]
11	*Arisaema rhizomatum* (雪里见/半截烂)	Rhizome	Detoxifying and relieving pain; Dispelling wind and eliminating dampness	Flavonoids and steroids	CIA mice	TNF-α, IL-1, IL-6, IL-33 ↓	-	[108]
12	*Laportea bulbifera* (红活麻)	Roots	Dispelling wind and eliminating dampness; Activating blood circulation and relieving pain	Coumarins	CIA mice	IFN-γ, IL-2, MHC-II CD86, IL-12 p70 ↓ IL-10 ↑	-	[109]

## Data Availability

No new data were created or analyzed in this study.

## Supplementary Materials

The following supporting information can be downloaded at: https://www.mdpi.com/article/10.3390/ph19060937/s1, Table S1. The used parts and species of the herbal drugs.

## References

[1] Radu A.F., Bungau S.G. (2021). Management of rheumatoid arthritis: An overview. Cells.

[2] Otón T., Carmona L. (2019). The epidemiology of established rheumatoid arthritis. Best Pract. Res. Clin. Rheumatol..

[3] Van der Woude D., Van der Helm-van Mil A.H.M. (2018). Update on the epidemiology, risk factors, and disease outcomes of rheumatoid arthritis. Best Pract. Res. Clin. Rheumatol..

[4] Smolen J.S., Aletaha D. (2015). Rheumatoid arthritis therapy reappraisal: Strategies, opportunities and challenges. Nat. Rev. Rheumatol..

[5] Roodenrijs N.M.T., Van der Goes M.C., Welsing P.M.J.W., Tekstra J., Lafeber F.P.J.G., Jacobs J.W.G., Van Laar J.M. (2020). Difficult-to-treat rheumatoid arthritis: Contributing factors and burden of disease. Rheumatology.

[6] Jurgens M.S., Jacobs J.W.G., Bijlsma J.W.J. (2011). The use of conventional disease-modifying anti-rheumatic drugs in established RA. Best Pract. Res. Clin. Rheumatol..

[7] Wang X., Kong Y.Q., Li Z.G. (2024). Advantages of Chinese herbal medicine in treating rheumatoid arthritis: A focus on its anti-inflammatory and anti-oxidative effects. Front. Med..

[8] Wang S., Zhang J., Liu W., Zhang L., Li R., Wang Y., Li S., Li L., Li J., Zhou M. (2025). Signal pathways in the treatment of rheumatoid arthritis with traditional Chinese medicine. J. Ethnopharmacol..

[9] Zhang Y.Q., Mao X., Li W.J., Chen W.J., Wang X.Y., Ma Z.C., Lin N. (2021). *Tripterygium wilfordii*: An inspiring resource for rheumatoid arthritis treatment. Med. Res. Rev..

[10] Tang Y.J., Liu Q.P., Feng Y.X., Zhang Y., Xu Z.H., Wen C.P., Zhang Y. (2020). Tripterygium Ingredients for Pathogenicity Cells in Rheumatoid Arthritis. Front. Pharmacol..

[11] Shen F.Y., Liu Y., Su W., Huang J.J., Peng C.Y., Li B., Wang W. (2015). Research on the Tujia ethnomedicine “seven” drugs. J. Hunan Univ. Chin. Med..

[12] Long F.X., Tang D.X. (2012). Discussion on the theory of disease caused by poison gas and the three-element theory of Tujia medicine. J. Jiangxi Univ. Tradit. Chin. Med..

[13] Dashtdar M., Dashtdar M.R., Dashtdar B., Kardi K., Shirazi M.K. (2016). The concept of wind in traditional Chinese medicine. J. Pharmacopunct..

[14] Lü S.W., Wang Q.S., Li G.Y., Sun S., Guo Y.Y., Kuang H.X. (2015). The treatment of rheumatoid arthritis using Chinese medicinal plants: From pharmacology to potential molecular mechanisms. J. Ethnopharmacol..

[15] Liu W.X., Jiang Q. (2023). Research progress on damp heat syndrome of rheumatoid arthritis. Zhonghua Zhongyi Yao Zazhi.

[16] Wang Z.Z., Fang Y.F., Wang Y., Mu F.X., Chen J., Zou Q.H., Zhong B., Li J.Y., Bo G.P., Zhang R.H. (2012). Logistic regression analysis of damp-heat and cold-damp impeding syndrome of rheumatoid arthritis: A perspective in Chinese medicine. Chin. J. Integr. Med..

[17] Hu M.Z., Su S.Y., Xue C.X., Zhang L., Zhang S.G., Yu H. (2018). Studies on medication laws towards rheumatoid arthritis based on analysis and comparison of medicine literatures. World Sci. Technol.Mod. Tradit. Chin. Med..

[18] Ma Q., Jiang J.G. (2016). The functional components from nature-derived drugs for the treatment of rheumatoid arthritis. Curr. Drug Targets.

[19] Wang J., Zhu F.Y., Huang W., Chen Z.Y., Zhao P., Lei Y.T., Liu Y.M., Liu X.J., Sun B., Li H.L. (2022). Therapeutic effect and mechanism of acupuncture in autoimmune diseases. Am. J. Chin. Med..

[20] Chen L.Q., Zhang J.H. (2007). Basic characteristics, research status, and development ideas of Tujia medicine. J. Hubei Minzu Univ. (Nat. Sci. Ed.).

[21] Nan Y.Y., Lin H.J., Hao R.X., Yuan L. (2019). An overview of Tujia medicine in the treatment of rheumatoid arthritis. Chin. J. Naturop..

[22] Guo Q., Wang Y.X., Xu D., Nossent J., Pavlos N.J., Xu J.K. (2018). Rheumatoid arthritis: Pathological mechanisms and modern pharmacologic therapies. Bone Res..

[23] McInnes I.B., Schett G. (2011). The pathogenesis of rheumatoid arthritis. N. Engl. J. Med..

[24] Deane K.D., Holers V.M. (2021). Rheumatoid arthritis pathogenesis, prediction, and prevention: An emerging paradigm shift. Arthritis Rheumatol..

[25] Mueller A.L., Payandeh Z., Mohammadkhani N., Mubarak S.M.H., Zakeri A., Alagheband Bahrami A., Brockmueller A., Shakibaei M. (2021). Recent advances in understanding the pathogenesis of rheumatoid arthritis: New treatment strategies. Cells.

[26] Lin Y.J., Anzaghe M., Schülke S. (2020). Update on the Pathomechanism, Diagnosis, and Treatment Options for Rheumatoid Arthritis. Cells.

[27] Mateen S., Zafar A., Moin S., Khan A.Q., Zubair S. (2016). Understanding the role of cytokines in the pathogenesis of rheumatoid arthritis. Clin. Chim. Acta.

[28] Ding Q., Hu W., Wang R., Yang Q.Y., Zhu M.L., Li M., Cai J.H., Rose P., Mao J.C., Zhu Y.Z. (2023). Signaling pathways in rheumatoid arthritis: Implications for targeted therapy. Signal Transduct. Target. Ther..

[29] Liu S., Ma H.X., Zhang H.X., Deng C.J., Xin P. (2021). Recent advances on signaling pathways and their inhibitors in rheumatoid arthritis. Clin. Immunol..

[30] Hu L., Liu R.J., Zhang L.L. (2022). Advance in bone destruction participated by JAK/STAT in rheumatoid arthritis and therapeutic effect of JAK/STAT inhibitors. Int. Immunopharmacol..

[31] Prajapati P., Doshi G. (2023). An update on the emerging role of Wnt/β-catenin, SYK, PI3K/AKT, and GM-CSF signaling pathways in rheumatoid arthritis. Curr. Drug Targets.

[32] Li J., Tang R.S., Shi Z., Li J.Q. (2020). Nuclear factor-κB in rheumatoid arthritis. Int. J. Rheum. Dis..

[33] Zheng Y.X., Wei K., Jiang P., Zhao J.N., Shan Y., Shi Y.M., Zhao F.Y., Chang C., Li Y.S., Zhou M. (2024). Macrophage polarization in rheumatoid arthritis: Signaling pathways, metabolic reprogramming, and crosstalk with synovial fibroblasts. Front. Immunol..

[34] Ralph J.A., Morand E.F. (2008). MAPK phosphatases as novel targets for rheumatoid arthritis. Expert Opin. Ther. Targets.

[35] Xin P., Xu X.Y., Zhang H.X., Hu Y.Z., Deng C.J., Sun S.Q., Liu S., Zhou X.G., Ma H.X., Li X.L. (2023). Mechanism investigation of Duhuo Jisheng pill against rheumatoid arthritis based on a strategy for the integration of network pharmacology, molecular docking and in vivo experimental verification. Pharm. Biol..

[36] Qu P.D., Wang H.Y., Wang W., Du S.Y., Peng Z.R., Hu Q., Tang X.H. (2023). Efficacy and safety of Duhuo-Jisheng decoction in rheumatoid arthritis: A systematic review and meta-analysis of 42 randomized controlled trials. Medicine.

[37] Xie Y., Mai C.T., Zheng D.C., He Y.F., Feng S.L., Li Y.Z., Liu C.X., Zhou H., Liu L. (2021). Wutou decoction ameliorates experimental rheumatoid arthritis via regulating NF-kB and Nrf2: Integrating efficacy-oriented compatibility of traditional Chinese medicine. Phytomedicine.

[38] Shen P., Lin W.J., Huang Y., Ba X., Han L., Li T.T., Qin K., Chen Z., Tu S.H. (2025). Wutou decoction attenuates rheumatoid arthritis in rats through SIRT1-mediated deacetylation of the HMGB1/NF-κB pathway. J. Ethnopharmacol..

[39] Han L., Yan J.H., Li T.T., Shen P., Ba X., Lin W.J., Zhang R.Y., Yang Y.Y., Li Y.F., Li C.N. (2024). Wutou decoction alleviates arthritis inflammation in CIA mice by regulating Treg cell stability and Treg/Th17 balance via the JAK2/STAT3 pathway. J. Ethnopharmacol..

[40] Zhang Y.M., Jin H.Z., Jia W.Y., Liu Y.Q., Wang Y.R., Xue S.Y., Liu Y., Hao H.Q. (2025). Ermiao San attenuating rheumatoid arthritis via PI3K/AKT/mTOR signaling activate HIF-1α induced glycolysis. J. Ethnopharmacol..

[41] Liu H.D., Kong L., Cao D.D., Zhan X.N., Gao X., Sun H., Yan G.L., Zhao Q.Q., Han Y., Wang X.J. (2024). Efficacy and mechanism of the Ermiao San series of formulas for rheumatoid arthritis based on Chinmedomics strategy. Phytomedicine.

[42] Jiao W., Xu J., Wu D.B., Yu J.H., Zhang M.Y., Liu L.J., Chen G.X. (2023). Anti-proliferation and anti-migration effects of Yishen Tongbi decoction in experimental rheumatoid arthritis by suppressing SLC3A2/integrin β3 signaling pathways. Phytomedicine.

[43] Hu C.Q., Peng S.Q., Zhao L.Y., Li M.L., Liu M.Q., Xu Y.P., Chen G.X. (2021). Yishen-tongbi decoction inhibits excessive activation of B cells by activating the FcγRIIb/Lyn/SHP-1 pathway and attenuates the inflammatory response in CIA rats. Biomed. Pharmacother..

[44] Xu J., Zhang L., Xu Y.P., Yu J.H., Zhao L.Y., Deng H., Li M.L., Zhang M.Y., Lei X.J., Hu C.Q. (2023). Effectiveness of Yishen Tongbi decoction versus methotrexate in patients with active rheumatoid arthritis: A double-blind, randomized, controlled, non-inferiority trial. Phytomedicine.

[45] Wang T.T., Jia Q.Y., Chen T., Yin H., Tian X.T., Lin X., Liu Y., Zhao Y.J., Wang Y.J., Shi Q. (2020). Alleviation of synovial inflammation of Juanbi-Tang on collagen-induced arthritis and TNF-Tg mice model. Front. Pharmacol..

[46] Chen Q., Yang J.M., Chen H., Pan T., Liu P.W., Xu S.J. (2023). Inhibition Ras/MEK/ERK pathway: An important mechanism of Baihu Jia Guizhi Decoction ameliorated rheumatoid arthritis. J. Ethnopharmacol..

[47] Zhang Q., Peng W., Wei S.J., Wei D.N., Li R.L., Liu J., Peng L.Y., Yang S., Gao Y.X., Wu C.J. (2019). Guizhi-Shaoyao-Zhimu decoction possesses anti-arthritic effects on type II collagen-induced arthritis in rats via suppression of inflammatory reactions, inhibition of invasion & migration and induction of apoptosis in synovial fibroblasts. Biomed. Pharmacother..

[48] Zhou L., Chen T., Li Z. (2025). Modern applications and analysis of Duhuo Jisheng Tang in the treatment of knee osteoarthritis. J. Liaoning Univ. Tradit. Chin. Med..

[49] (2023). Standardization Project Team of “Clinical Application Guidelines for Chinese Patent Medicines in Dominant Diseases”. Clinical Application Guidelines for Chinese Patent Medicines in the Treatment of Rheumatoid Arthritis (2022). Chin. J. Integr. Tradit. West. Med..

[50] Wang X.D. (2011). Therapeutic Effects Research and Contribution on IL-8 and γIP-10 of Kunxian Capsule in Treating Rheumatoid Arthritis. Ph.D. Thesis.

[51] Lin C.S., Yang X.Y., Dai L., Sun W.F., Yang S.F., Shen Y., Xu Q., Wang X.D. (2011). Multicenter clinical study on therapeutic effect of Kunxian capsule on rheumatoid arthritis. Chin. J. Integr. Med..

[52] Ma R.J., Kannan M., Zhuang K.Y., Xia Q., Sun D., Tu P.F., Fan T.P., Liu K.C., Zhang Y. (2023). Pharmacological importance of Kunxian Capsule in clinical applications and its adverse effects: A review. Chin. Herb. Med..

[53] Lin N., Zhang Y.Q., Jiang Q., Liu W., Liu J., Huang Q.C., Wu K.Y., Tu S.H., Zhou Z.S., Chen W.H. (2021). Clinical practice guideline for *Tripterygium* glycosides/*Tripterygium wilfordii* tablets in the treatment of rheumatoid arthritis. Front. Pharmacol..

[54] Zhang M.Y. (2025). Study on the Mechanism of Action of Leigongteng Polyglucoside Tablets Combined with Methotrexate in the Treatment of Rheumatoid Arthritis. Master’s Thesis.

[55] Gao Y.H. (2023). Study on the Substances and Mechanism of the Efficacy and Hepatotoxicity of Tripterygium Glycosides Tablets. Master’s Thesis.

[56] Zheng W.H., Mei Y.F., Chen C.H., Cai L.Y., Chen H. (2021). The effectiveness and safety of *Tripterygium wilfordii* glycosides combined with disease-modifying anti-rheumatic drugs in the treatment of rheumatoid arthritis: A systematic review and meta-analysis of 40 randomized controlled trials. Phytother. Res..

[57] Liu L.L., Tian Y.G., Su X.H., Fan Y.F., Li C., Zhu X.X., Cao W., Liu T., Wang H.L., Xu Y. (2019). Comparative study on dose-toxicity-effect of Tripterygium Glycosides Tablets and *Tripterygium wilfordii* Tablets on CIA model rats. Chin. J. Chin. Mater. Med..

[58] Ba X., Wang H., Huang Y., Yan J.H., Han L., Lin W.J., Shen P., Huang Y., Yang S.S., Qin K. (2023). Simiao pill attenuates collagen-induced arthritis and bleomycin-induced pulmonary fibrosis in mice by suppressing the JAK2/STAT3 and TGF-β/Smad2/3 signalling pathway. J. Ethnopharmacol..

[59] Wang X.Y., Zhang X.L., Zhang L., Li Y.B. (2010). Effects and mechanisms of Simiao pill on adjuvant arthritis rats model. Chin. J. Chin. Mater. Med..

[60] Hu X.H. (2015). Clinical observation on simiao pills for rheumatoid arthritis in active stage. Chin. J. Clin. Med..

[61] Shan Y., Zhao J., Wei K., Jiang P., Xu L., Chang C., Xu L., Shi Y., Zheng Y., Bian Y. (2023). A comprehensive review of *Tripterygium wilfordii* Hook. f. in the treatment of rheumatic and autoimmune diseases: Bioactive compounds, mechanisms of action, and future directions. Front. Pharmacol..

[62] Ru Y., Luo Y., Zhou Y., Kuai L., Sun X., Xing M., Liu L., Lu Y., Hong S., Chen X. (2019). Adverse events associated with treatment of *Tripterygium wilfordii* Hook F: A quantitative evidence synthesis. Front. Pharmacol..

[63] Chan Y.T., Wang N., Feng Y. (2021). The toxicology and detoxification of *Aconitum*: Traditional and modern views. Chin. Med..

[64] Lippert A., Renner B. (2022). Herb-drug interaction in inflammatory diseases: Review of phytomedicine and herbal supplements. J. Clin. Med..

[65] Li S., Chen Q., Zhang Y., Wang D., Hu H., Li J., Zhang C., Zhang J. (2024). Hyaluronic acid dissolving microneedle patch-assisted acupoint transdermal delivery of triptolide for effective rheumatoid arthritis treatment. Sci. Rep..

[66] Liang M.G., Yan L., Mei Z.G., Luo Y.N., Hou X.Q., Feng Z.T. (2020). Methodological and reporting quality evaluation of meta-analyses on the Chinese herbal preparation Zheng Qing Feng Tong Ning for the treatment of rheumatoid arthritis. BMC Complement. Med. Ther..

[67] Li C., Yao Y., Wang Y., Sun L.Y., Ding C.Z. (2012). Effects of Zhengging Fengtongning combined with methotrexate on the expression of RANKL and MMPs in the serum of collagen induced arthritis rats. J. Nanjing Univ. TCM.

[68] Wang K., Zhang D.M., Liu Y., Wang X., Zhao J.T., Sun T.T., Jin T.T., Li B.L., Pathak J.L. (2018). Traditional Chinese medicine formula Bi-Qi capsule alleviates rheumatoid arthritis-induced inflammation, synovial hyperplasia, and cartilage destruction in rats. Arthritis Res. Ther..

[69] Wang Z., Wu J.Q., Li D.Y., Tang X., Zhao Y., Cai X., Chen X.H., Chen X.M., Huang Q.C., Huang R.Y. (2020). Traditional Chinese medicine Biqi capsule compared with leflunomide in combination with methotrexate in patients with rheumatoid arthritis: A randomized controlled trial. Chin. Med..

[70] Xu Y.M., Rong X.F., Tan H.F., Peng F.F. (2015). Effects of Chinese medicine BiOi capsule on the expressions of JAK/STAT signal pathways in CIA rats. Immunol. J..

[71] Li G., Du S.M., Yan S.Y., Wang Y., Bu R.Z., Cheng M.F., Zhang Y., Chen Q., Wu Y.Z., Zhang X.Q. (2025). Mechanism of Biqi capsules in the treatment of gout based on network pharmacology and experimental verification. J. Ethnopharmacol..

[72] Xia Y. (2021). Study on the Mechanism of Medicated Serum Containing Yishen Juanbi Pills in Inhibiting Osteoclast Differentiation and Function Based on the JAK2/STAT3 Signaling Pathway. Master’s Thesis.

[73] Xue H.W., Zhao H.Y., Ma G.J. (2022). Clinical effect of Yishen Juanbi Pills combined with methotrexate tablets in the treatment of rheumatoid arthritis. China Med. Her..

[74] Wang J.J., Liu J., Pei T.X., Guo J.Y., Guo C.M., Yang D., Zhang Z.P., Shen X.P. (2016). Study on effects of Yishen Juanbi Pill on histopathologic changes in ankle joint of type II collagen induced arthritis in rats and its mechanism. Chin. Tradit. Herb Drugs.

[75] Zheng H.L. (2022). Study on the Mechanism of Wangbi Tablets Regulating the Wnt/β-catenin Pathway to Intervene in Bone Formation in Ovariectomized CIA Rats. Master’s Thesis.

[76] Wu J.L., Su L., Huang Q.C., Wang W.P., Su X., Wang Y., Zhai F.M., Liu Y.W. (2024). Wangbi Capsule for active rheumatoid arthritis: A multicenter, randomized, double-blind, parallel-controlled study. Chin. J. Integr. Med..

[77] Shu H.Y., Shi Y.J., Li L., Zhao N., Lu C., Lu A.P., He X.J. (2021). Dissecting the molecular mechanism of Wang-Bi Capsule in the treatment of experimental rheumatoid arthritis based on synovial tissue proteomic analysis. J. Immunol. Res..

[78] Yuan B., Su X.H., Guo W.Y., Wang Q., Liu C.F., Han L., Lin N. (2021). Effect and mechanism of Yuxuebi Tablet against collagen-induced arthritis of rats. Chin. J. Exp. Tradit. Med. Formul..

[79] Su X.H., Yuan B., Tao X.Y., Guo W.Y., Mao X., Wu A.G., Wang Q., Liu C.F., Zhang Y.Q., Kong X.Y. (2022). Anti-angiogenic effect of YuXueBi tablet in experimental rheumatoid arthritis by suppressing LOX/Ras/Raf-1 signaling. J. Ethnopharmacol..

[80] Chen W.J., Zhang C., Xu M.Z., Li T., Li X., Li P.H., Gong X., Qu Y., Zhou C.L., Mao X. (2025). Yu-Xue-Bi capsule ameliorates aggressive synovitis and joint damage in rheumatoid arthritis via modulating the SUCNR1/HIF-1α/TRPV1 axis. Phytomedicine.

[81] Xue J.L., Zhao X.Y., Jia F., Han T.T., Guo L.L. (2025). Effect and mechanism of Panlongqi Tablet on rats with rheumatoid arthritis. Chin. J. Osteoporos..

[82] Niu H.H., Jiao Y.C., Bai R.X. (2022). Study on the clinical efficacy of Panlongqi Tablets in treating rheumatoid arthritis with rheumatoid arthralgia syndrome. J. China Prescr. Drug.

[83] Niu X.F., Yang Y.J., Yu J.J., Song H.X., Yu J.B., Huang Q.X., Liu Y., Zhang D.Z., Han T.F., Li W.F. (2023). Panlongqi tablet suppresses adjuvant-induced rheumatoid arthritis by inhibiting the inflammatory reponse in vivo and in vitro. J. Ethnopharmacol..

[84] Niu X.F., Yang Y.J., Yu J.J., Song H.X., Yu J.B., Huang Q.X., Liu Y., Zhang D.Z., Han T.F., Li W.F. (2009). Regulation effect of Shi-re-bi tablet on T Lymphocyte and cytokine network in the adjuvant-induced arthritis rats. Chin. J. Exp. Tradit. Med. Formul..

[85] Lv X.J., Liu J., Lu J., Wang C.F., Nan S.H., Shen X.P. (2013). Anti-inflammatory effect and mechanism of Xinhuang tablet. Tianjin J. Tradit. Chin. Med..

[86] Yang G.Q., Wu Y., Kang J.Y., Sun Q. (2022). Effects of Xinhuang tablets external application combined with Du Moxibustion on rheumatoid arthritis. World Clin. Drugs.

[87] Yang Y.P., Hussain N., Zhang L., Jia Y.Z., Jian Y.Q., Li B., Iqbal Choudhary M., Rahman A.u., Wang W. (2020). Kadsura coccinea: A rich source of structurally diverse and biologically important compounds. Chin. Herb. Med..

[88] Yuan H.W., Su W., Wang M.Y., Liu S.Q., Yu H.H., Li B., Yang Y.P., Wang W. (2025). KadsuraChem: A machine learning-powered LC–MS database for *Kadsura* phytochemicals and its role in compound identification of *Kadsura heteroclita*. J. Agric. Food. Chem..

[89] Wang M.Y., Jiang S., Yuan H.W., Zafar S., Hussain N., Jian Y.Q., Li B., Gong L.M., Peng C.Y., Liu C.X. (2021). A review of the phytochemistry and pharmacology of *Kadsura heteroclita*, an important plant in Tujia ethnomedicine. J. Ethnopharmacol..

[90] Deng Y.S., Chen Y.X., Zheng H., Li B., Liang L., Su W., Ahmad B., Yang Y.P., Yuan H.W., Wang W. (2025). Xuetongsu ameliorates synovial inflammatory hyperplasia in rheumatoid arthritis by inhibiting JAK2/STAT3 and NF-κB signaling pathways. J. Ethnopharmacol..

[91] Yu H.H., Lin Y., Zeng R., Li X., Zhang T., Tasneem S., Chen C., Qiu Y.X., Li B., Liao J. (2019). Analgesic and anti-inflammatory effects and molecular mechanisms of *Kadsura heteroclita* stems, an anti-arthritic Chinese Tujia ethnomedicinal herb. J. Ethnopharmacol..

[92] Yu H.H., Zeng R., Lin Y., Li X., Tasneem S., Yang Z., Qiu Y.X., Li B., Wang Y.H., Cai X. (2019). *Kadsura heteroclita* stem suppresses the onset and progression of adjuvant-induced arthritis in rats. Phytomedicine.

[93] Yang Y.P., Liu Y.B., Yu H.H., Xie Q.L., Wang B., Jiang S., Su W., Mao Y., Li B., Peng C.Y. (2022). Sesquiterpenes from *Kadsura coccinea* attenuate rheumatoid arthritis-related inflammation by inhibiting the NF-κB and JAK2/STAT3 signal pathways. Phytochemistry.

[94] Yang Y.P., Jian Y.Q., Liu Y.B., Ismail M., Xie Q.L., Yu H.H., Wang B., Li B., Peng C.Y., Liu B. (2021). Triterpenoids from *Kadsura coccinea* with their anti-inflammatory and inhibited proliferation of rheumatoid arthritis-fibroblastoid synovial cells activities. Front. Chem..

[95] Zhou K., Yu H.H., Duan H.H., Yang Y.P., Sheng W.B., Qiu Y.X., Wang W., Li B. (2025). Total saponins of *Panax japonicus* alleviate adjuvant-induced arthritis in rats by regulating macrophage polarization and intestinal microbiota. J. Ginseng Res..

[96] Xiang Y., Yuan L. (2021). Compendium of Anti-Rheumatic Tujia Ethnomedicines.

[97] Xiang Y., Zhang T.T., Yin C.P., Zhou J.W., Huang R., Gao S.S., Zheng L.M., Wang X.G., Manyande A., Tian X.B. (2016). Effects of the stem extracts of *Schisandra glaucescens* Diels on collagen-induced arthritis in Balb/c mice. J. Ethnopharmacol..

[98] Wu W.M., Ruan H.L. (2019). Triterpenoids and lignans from the stems of Schisandra glaucescens. Nat. Prod. Res..

[99] Qin H.G., Fu Y.L., Zhou K., Song H.H., Fang G., Chen Q., Pang Y.Z. (2023). *Toddalia asiatica* extract attenuates adjuvant-induced arthritis by modulating colon Th17/Treg balance and colony homeostasis. J. Ethnopharmacol..

[100] Yan M.Y., Sun Y.J., Ding L., Sun J.X., Song J.Z., Zhou W.B., Pei L.P. (2022). Three new triterpenoid saponins from *Aralia echinocaulis*. Chin. Herb. Med..

[101] Li Y.Z., He N., Shen L.Q., Liu M.D. (2019). Apoptotic effect of *Aralia echinocaulis* extract on fibroblast-like synoviocytes in rats with adjuvant-induced arthritis via inhibiting the Akt/Hif-1α signaling pathway in vitro. J. Pharmacol. Sci..

[102] Wu Z., Xia J.H., Guo Z.Y., Lei J.C., Yu J.Q. (2021). Biological activities of the extracts and compounds from the bark of *Pterocarya hupehensis* Skan. Nat. Prod. Res..

[103] Zhu J., Wu L.L., Zhang Q.S., Wang X.P., Yuan L., Xiang Y. (2019). Therapeutic effect of ethanol extract of *Pterocarya hupehensis* Skan on collagen-induced arthritis rats and its mechanism. Chin. Pharmacol. Bull..

[104] Sun X.B., Li W., Rao Y.S., Mao C.X., Xiang D.X. (2023). Effect of total saponins from *Clematis henryi* Oliv. on the activation of synovial cells and related mechanism. Cent. South Pharm..

[105] Chen Y., Yang H., Lu Y.X., Zhou C.X., Yan M.Y., Li L.J., Hu Z.H. (2021). Purification of total flavonoids from the leaves of *llex centrochinensis* and their anti-rheumatoid arthritis synovial inflammation. J. Hubei Univ..

[106] Liang C.J., Yin J.T., Ma Y.L., Zhang X., Zhang L.T. (2020). Quantitative determination of characteristic components from compound of *Lysionotus pauciflorus* Maxim. by LC–MS/MS and its application to a pharmacokinetic study. J. Pharm. Biomed. Anal..

[107] Fu Z.W. (2016). The Effect and Mechanism Study of *Lysionotus pauciflorus* Maxim in Tujia Medicine on Rat Model with Adjuvant Arthritis. Master’s Thesis.

[108] Chen C.X., Zhang P., Pi H.F., Ruan H.L., Hu Z.H., Wu J.Z. (2011). Extracts of *Arisaema rhizomatum* C.E.C. Fischer attenuate inflammatory response on collagen-induced arthritis in BALB/c mice. J. Ethnopharmacol..

[109] Luo X., Li L.L., Zhang S.S., Lu J.L., Zeng Y., Zhang H.Y., Xiang M. (2011). Therapeutic effects of total coumarins from *Urtica dentata* Hand on collagen-induced arthritis in Balb/c mice. J. Ethnopharmacol..

[110] Gamus D., Shoenfeld Y. (2025). Acupuncture therapy in autoimmune diseases: A narrative review. Autoimmun. Rev..

[111] Wan R., Fan Y., Zhao A., Xing Y., Huang X., Zhou L., Wang Y. (2022). Comparison of efficacy of acupuncture related therapy in the treatment of rheumatoid arthritis: A network meta-analysis of randomized controlled trials. Front. Immunol..

[112] Liao C., Tao S., Xiong Y., Dai J., Bai Y., Wang X., Li Y., Wu P. (2023). The effects and potential mechanisms of moxibustion for rheumatoid arthritis related pain: A randomized, controlled trial. J. Pain Res..

[113] Ren D.D., Ma X., Wang W.J., Zhao Y. (2022). Application of Chinese herbal fumigation combined with Tuina in patients with early-stage rheumatoid arthritis. Chin. Gen. Pract. Nurs..

[114] Zhang Y.D., Xu J., Yuan D.P., Yuan L. (2018). Research status of Tujia ethnomedicine in the treatment of rheumatoid arthritis (RA). J. Hubei Univ. Natl..

