# Supplementary material for: Pharmacological Exploration of Traditional Chinese Medicine and Tujia Ethnomedicine in Rheumatoid Arthritis Therapy: From Historical Clinical Wisdom to Contemporary Scientific Inquiry
Source: Pharmaceuticals (Basel). 2026 Jun 14;19(6):937. doi: 10.3390/ph19060937 (PMC13304939; doi:10.3390/ph19060937)
Supplement: Supplementary file 1 [file pharmaceuticals-19-00937-s001.zip › pharmaceuticals-4300419-Supplementary.pdf]

Supplementary data

Pharmacological exploration of Traditional Chinese Medicine and Tujia ethnomedicine in rheumatoid arthritis therapy: From historical clinical wisdom to contemporary scientific inquiry

Qingling Xie[†], Jisheng Liu[†], Wei Su, Jiangyi Luo, Mengying Lyu, Yan Zhao, Yunmei Lan, Ling Liang, Caiyun Peng, Wei Wang*, Hanwen Yuan*

TCM and Ethnomedicine Innovation & Development International Laboratory, School of Pharmacy, Hunan University of Chinese Medicine, Changsha, 410208, China.

** Corresponding author at: TCM and Ethnomedicine Innovation & Development International Laboratory, School of Pharmacy, Hunan University of Chinese Medicine, Changsha, 410208, China.*

E-mail addresses: wangwei402@hotmail.com (W. Wang), Hanwyuan@hnucm.edu.cn (H. Yuan).

† These authors contributed equally to this study.

Table S1. The used parts and species of the herbal drugs.

No.	Herbal drugs	Used parts	Species
1	Duhuo (独活)	root	*Angelica pubescens* Maxim.
2	Xixin (细辛)	flower bud	*Magnolia biondii* Pamp., *Magnolia denudata* Desr., or *Magnolia sprengeri* Pamp.
3	Rougui (肉桂)	bark	*Cinnamomum cassia* Presl
4	Fangfeng (防风)	root	*Saposhnikovia divaricata* (Turcz.) Schischk.
5	Qinjiao (秦艽)	root	*Gentiana macrophylla* Pall., *Gentiana straminea* Maxim., *Gentiana crassicaulis* Duthie ex Burk., or *Gentiana dahurica* Fisch.
6	Duzhong (杜仲)	bark	*Eucommia ulmoides* Oliv.
7	Niuxi (牛膝)	root	*Achyranthes bidentata* Bl.
8	Sangjisheng (桑寄生)	Stem branches with leaves	*Taxillus chinensis* (DC.) Danser
9	Dihuang (地黄)	root	*Rehmannia glutinosa* Libosch.
10	Shaoyao (芍药)	root	*Paeonia lactiflora* Pall.
11	Danggui (当归)	root	*Angelica sinensis* (Oliv.) Diels
12	Chuanxiong (川芎)	rhizome	*Ligusticum chuanxiong* Hort.
13	Renshen (人参)	root and rhizome	*Panax ginseng* C. A. Mey.
14	Fuling (茯苓)	sclerotia	*Poria cocos* (Schw.) Wolf
15	Gancao (甘草)	root and rhizome	*Glycyrrhiza uralensis* Fisch., *Glycyrrhiza inflata* Bat., or *Glycyrrhiza glabra* L.
16	Chuanwu (川乌)	root	*Aconitum carmichaelii* Debx.
17	Mahuang (麻黄)	stem	*Ephedra sinica* Stapf, *Ephedra intermedia* Schrenk et C. A. Mey., *Ephedra equisetina* Bge.
18	Huangqi (黄芪)	rhizome	*Astragalus membranaceus* (Fisch.) Bge.
19	Huangbai (黄柏)	bark	*Phellodendron chinense* Schneid.
20	Cangzhu (苍术)	rhizome	*Atractylodes lancea* (Thunb.) DC. or *Atractylodes chinensis* (DC.) Koidz.
21	Kunming Shanhaitang (昆明山海棠)	root	*Tripterygium hypoglaucum* (Devl.) Hutch.
22	Gouqizi (枸杞子)	fruit	*Lycium barbarum* L.
23	Nüzhenzi (女贞子)	fruit	*Ligustrum lucidum* Ait.
24	Mohanlian (墨旱莲)	aerial parts	*Eclipta prostrata* L.
25	Danshen (丹参)	root and rhizome	*Salvia miltiorrhiza* Bge.
26	Qianghuo (羌活)	root and rhizome	*Notopterygium incisum* Ting ex H. T. Chang or *Notopterygium franchetii* H. de Boiss.
27	Guizhi (桂枝)	branch	*Cinnamomum cassia* Presl
28	Baishao (白芍)	root	*Paeonia lactiflora* Pall.
29	Shigao (石膏)	gypsum	-
30	Zhimu (知母)	rhizome	*Anemarrhena asphodeloides* Bge.
31	Jingmi (粳米)	japonica rice	-
32	Shengjiang (生姜)	rhizome	*Zingiber officinale* Rosc.
33	Baizhu (白术)	rhizome	*Atractylodes macrocephala* Koidz.
34	Fuzi (附子)	root	*Aconitum carmichaelii* Debx.
35	Yingyanghuo (淫羊藿)	leave	*Epimedium brevicornu* Maxim., *Epimedium sagittatum* (Sieb. et Zucc.) Maxim., *Epimedium pubescens* Maxim., or *Epimedium koreanum* Nakai
36	Tusizi (菟丝子)	seed	*Cuscuta australis* R. Br. or *Cuscuta chinensis* Lam.

37	Tripterygium Glycosides (雷公藤多苷)	Glycosides prepared from the root	*Tripterygium wilfordii* Hook.f.
38	Yiyiren (薏苡仁)	kernel	*Coix lacryma*-jobi L. var. mayuen (Roman.) Stapf
39	Maqianzi (马钱子)	seed	*Strychnos nuxvomica* L.
40	Dilong (地龙)	-	*Pheretima aspergillum* (E. Perrier), *Pheretima vulgaris* Chen, *Pheretima guillelmi* (Michaelsen), or *Pheretima pectinifera* Michaelsen
41	Dangsheng (党参)	root	*Codonopsis pilosula* (Franch.) Nannf., *Codonopsis pilosula* Nannf. var. *modesta* (Nannf.) L. T. Shen, or *Codonopsis tangshen* Oliv.
42	Sanqi (三七)	root and rhizome	*Panax notoginseng* (Burk.) F. H. Chen
43	Gusuibu (骨碎补)	rhizome	*Drynaria fortunei* (Kunze) J. Sm
44	Xuchangqing (徐长卿)	root and rhizome	*Cynanchum paniculatum* (Bge.)Kitag.
45	Tubiechong (土鳖虫)	-	*Eupolyphaga sinensis* Walker or *Polyphaga plancyi* Bolívar
46	Wugong (蜈蚣)	-	*Scolopendra subspinipes mutilans* L. Koch
47	Quanxie (全蝎)	-	*Buthus martensii* Karsch
48	Fengfang (蜂房)	hive	-
49	Wushaoshe (乌梢蛇)	-	*Ptyas dhumnades* (Cantor)
50	Yanhusuo (延胡索)	rhizome	*Corydalis yanhusuo* W. T. Wang
51	Luxiancao (鹿衔草)	whole plant	*Pyrola calliantha* H. Andres or *Pyrola decorata* H. Andres
52	Xungufeng (寻骨风)	whole plant	*Aristolochia mollissima* Hance
53	Laohecao (老鹳草)	aerial parts	*Erodium stephanianum* Willd., *Geranium wilfordii* Maxim., or *Geranium carolinianum* L.
54	Jixueteng (鸡血藤)	stem	*Spatholobus suberectus* Dunn
55	Lvcao (律草)	whole plant	*Humulus scandens* (Lour.) Merr.
56	Huzhang (虎杖)	root and rhizome	*Polygonum cuspidatum* Sieb. et Zucc.
57	Xuduan (续断)	root	*Dipsacus asper* Wall. ex Henr
58	Weilingxian (威灵仙)	root and rhizome	*Clematis chinensis* Osbeck, *Clematis hexapetala* Pall., or *Clematis manshurica* Rupr.
59	Zaoci (皂刺)	thorn	*Gleditsia sinensis* Lam.
60	Yangu (羊骨)	bones from goat or sheep	-
61	Shenjincao (伸筋草)	whole plant	*Lycopodium japonicum* Thunb.
62	Honghua (红花)	flower	*Carthamus tinctorius* L.
63	Ruxiang (乳香)	resin	*Boswellia carterii* Birdw. or *Boswellia bhaw-dajiana* Birdw.
64	Moyao (没药)	resin	*Commiphora myrrha* Engl. or *Commiphora molmol* Engl.
65	Chuanniuxi (川牛膝)	root	*Cyathula officinalis* Kuan
66	Jianghuang (姜黄)	rhizome	*Curcuma longa* L.
67	Xiangfu (香附)	rhizome	*Cyperus rotundus* L.
68	Panlongqi (盘龙七)	rhizome	*Bergenia scopulosa* T. P. Wang
69	Caowu (草乌)	root	*Aconitum kusnezoffii* Reichb.
70	Tiebangchui (铁棒锤)	root	*Aconitum pendulum* Busch
71	Wujiapi (五加皮)	root bark	*Aconitum kusnezoffii* Reichb.

72	Guoshanlong (过山龙)	root bark	*Ampelopsis aconitifolia* Bunge
73	Rendongteng (忍冬藤)	stem and branches	*Lonicera japonica* Thunb.
74	Lianqiao (连翘)	fruit	*Forsythia suspensa* (Thunb.) Vahl
75	Fenbixie (粉萆薢)	rhizome	*Dioscorea hypoglauca* Palibin
76	Sangzhi (桑枝)	branch	*Morus alba* L.
77	Fangji (防己)	root	*Stephania tetrandra* S. Moore
78	Zhongjiefeng (肿节风)	whole plant	*Sarcandra glabra* (Thunb.)Nakai
79	Niuhuang (牛黄)	bezoar	-
80	Zhudanfen (猪胆粉)	the biliary powder from pig	-
81	Xiaofantianhua (肖梵天花)	root or whole plant	*Urena lobata* Linn.
82	Zhenzhu (珍珠)	pearl	-
83	Shuiniujiao Nongsuofen (水牛角浓缩粉)	concentrated extract powder of the corner	*Bubalus bubalis* Linnaeus
84	Hongqu (红曲)	red yeast rice	-
85	Zhuangjindan (壮筋丹)	root	*Silene tatarinowii* Regel
86	Zhuzishen (珠子参)	rhizome	*Panax japonicus* C. A. Mey. var. major (Burkill) C. Y. Wu et K. M. Feng or *Panax japonicu*s C. A. Mey. var. bipinnatifidus （Seem.）C. Y. Wu et K. M. Feng 的
87	Qingwaqi (青蛙七)	rhizome	*Iris tectorum* Maxim.
88	Muxiang (木香)	root	*Aucklandia lappa* Decne.
89	Zusima (祖司麻)	root bark/stem bark	*Daphne giraldii* Nitsche
90	Luoshiteng (络石藤)	stem and branch	*Trachelospermum jasminoides* (Lindl.) Lem.
91	Baimaoqi (白毛七)	root and rhizome	Chloranthus multistachys Pei.
92	Laoshuqi (老鼠七)	root	Hylomecon japonica (Thunb.) Prantl
93	Zhizhuliao (支柱蓼)	rhizome	*Polygonum suffultum* Maxim.
94	Zhugenqi (竹根七)	rhizome	*Disporopsis fuscopicta* Hance
95	Xiecao (缬草)	root and rhizome	*Valeriana officinalis* L.
96	Yangjiaoqi (羊角七)	rhizome	*Bletilla striata* (Thunb). Reichb. f.
97	Balima (八里麻)	rhizome	*Sambucus chinensis* Lindl.
98	Chonglou (重楼)	rhizome	*Paris polyphylla* Smith var. chinensis (Franch.) Hara or *Paris polyphylla* Smith var. yunnanensis (Franch.) Hand.-Mazz.
